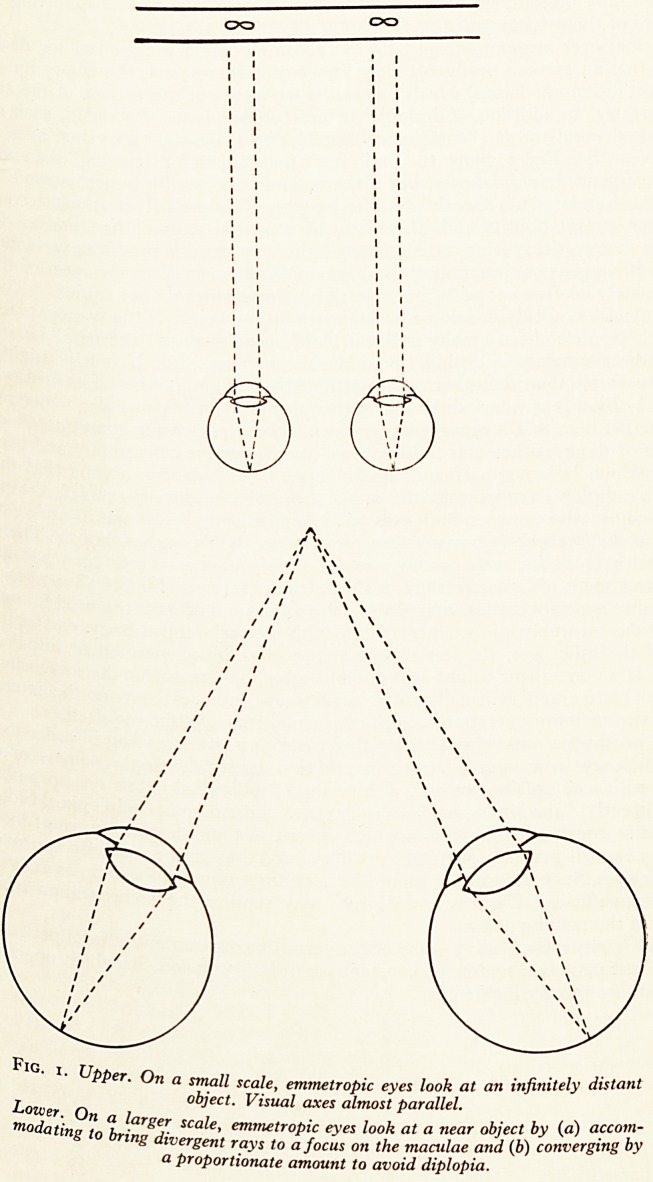# Squint

**Published:** 1959-04

**Authors:** Calbert I. Phillips

**Affiliations:** Ophthalmologist, United Bristol Hospitals.


### SQUINT

BY

CALBERT I. PHILLIPS,
*Ophthalmologist, United Bristol Hospitals.*

The investigation and treatment of strabismus take up a high proportion of t ophthalmologist's time. Its treatment is rewarding for the patients are often child in whom the prevention of amblyopia, restoration of binocular vision and impro ment in appearance will be of great benefit for the remainder of their lives. T assessment of a diplopic patient is an interesting exercise and may contribute much a neurological diagnosis.

The patient, if adult, usually complains of blurred or double vision. A great deal time and trouble are often saved if uniocular diplopia be excluded from the st If his blurred vision disappears when he closes each eye separately, then his diplo is binocular. Many patients with astigmatism or early cataract complain of "dou vision" (this, in a sense, is true, though "blurred vision" would be more corre which, of course, remains in the affected eye(s) when the other is closed. The pati can try out this test on the spot so that binocular and uniocular diplopia can immediately differentiated.

The first aim in diagnosis is to decide whether a patient has an (a) incomitant (b) concomitant squint. Usually the former are adults complaining of double visi and the latter children. The term "incomitant" implies that the visual axes of the t eyes are not *both* pointing towards the object of attention *in all directions* of g A paralysis or paresis of, say, the right lateral rectus muscle will result in mis-alignm when the patient looks to the right and therefore diplopia: there is no abnormal in the other directions. The term "concomitant" strabismus means that the vis axes maintain the same, abnormal, relation to each other in all directions of g The visual axis can be regarded as a line drawn from the macula through the cen of the pupil.

### COVER TEST

Before history taking, aetiology and mechanisms are discussed, it is convenient describe this important part of the examination. The cover test proper is an object means of determining the presence of squint and is particularly useful for childr with concomitant squints. Various refinements are used but the form to be describ is the basic one for the detection of a manifest squint—whether it is concomitant incomitant. See Plate XIX. Tell the patient to look at an object e.g. the tip of a pen held at about arm's length from his eyes, and to keep looking at it. Now cover the ri eye, but watch the left; if the left does not move, it is not, and was not, squinti Next uncover the right eye and tell the patient to look at the pen again (both e being uncovered). Then cover the left eye but watch the right; if the latter does n move to take up fixation, no manifest squint is present—in either eye. The young the child the less he will co-operate and often the interpretation of the test is difficu A child's interest may be maintained if he is told to look at a flashing torch light o small brightly coloured toy. Each eye should have at least fair visual acuity—a bli eye will not move to take up fixation though it may be squinting.

Often, in spite of a negative cover test, observant parents may insist that the chi has a squint. Either the squint is intermittent or some other condition simulates The usual cause of "apparent squint" is epicanthic folds at the inner canthi whi produce an appearance of convergence of the eyes—see Plate XX.

(*a*)

(*b*)

(*c*)

(*d*)

FIG. 1. Cover test. *(a) The patient is told to look at a small object held in the examiner's hand about a yard away. (b) The right eye is covered and the examiner notes that the left eye moves outwards to take up fixation i.e. the left eye has a convergent squint. (c) Both eyes are uncovered again and object is re-fixed. (d) The left eye is covered but no movement occurs in the right eye i.e. it does not squint.*

Diagnosis. *Left convergent squint. Since movements of both eyes were full, "concomitance" is proved. The squint was "cured" by the correction of the hypermetropia with glasses.*

PLATE XX

FIG. 2. *Epicanthic folds simulating convergent squint. Cover test negative.*

### I. INCOMITANT STRABISMUS

Careful history-taking is as vital in ophthalmology as in any other branch of medicine.

Its duration and possible intermittency having been ascertained, the next important question is whether diplopia is vertical or horizontal. "Are the two images side by side or one above the other—or both?" "Does the separation increase when you look right, left, up or down; near or far?" From this information, if the patient is observant, a good indication of the affected muscle can be obtained. Horizontal diplopia only to the right suggests a (right) abducent nerve palsy, common in elderly people, which often recovers within a few weeks and is usually attributed to a "vascular" cause—a rather vague explanation which implies either pressure on the nerve by an atheromatous intracranial artery or blockage of a nutrient vessel. These symptoms *could* be due to a left medial rectus palsy but in isolation this is rare, even in disseminated sclerosis.

Ocular movements should be examined. It is easier to assess the range of movement of each eye separately, the one not under observation being covered by the examiner's hand or an envelope etc. Very often a muscle may be only paretic so that no obvious impairment of movement exists. Clinically, the affected muscle may be discovered by the following system. The patient is asked to look at, say, a pen nib with both eyes open and to watch it as it is moved upwards, downwards, to his right and left, and also to the intermediate points. The area in which maximum separation of the doubled images occurs is ascertained. Suppose the separation to be vertical, and maximal downwards and to the (patient's) left. Which eye and which muscle is affected? The image which is further away from the centre (the lower in this position) always belongs to the paretic eye so. . . . To the patient looking at the pen nib down and to his left: "Close your left eye and tell me which image disappears". . . . "The upper". Therefore the *right* is the affected eye since the further-away-from-centre image belongs to it. Now, the main action of the superior oblique muscle is depression of the eye when it is already adducted towards the nose, therefore the right superior oblique is the affected muscle—a common occurrence.

However, often the results are equivocal, and the ingenious "Hess screen" examination is very useful in such cases. Over the patient's eyes are placed goggles, a red glass before the right and a green before the left. He is seated one metre from a black screen over which are scattered, at regular intervals, red dots or red lights. Now, the right eye can see these *red* dots but the left cannot (because the green glass allows only green rays to pass, not red). Into his hand he is given a pointer with a green ring at its tip and he is told to place the green ring (see only by the left eye) round the red dots. If he has, say, a left lateral rectus paresis, the right eye will look at each red dot but, when the gaze is directed towards the patient's left, the left eye will not have moved far enough and therefore the green tip of his pointer will be placed some distance short of the dots in that region. However, when he looks up, down and to the right, his eyes will be "comitant" and he will place the green ring over the red dots successfully. A chart is completed from which can be seen at a glance the direction in which one eye falls short and therefore which muscle is paretic.

Once the weak or paralysed muscle has been detected, the cause remains to be diagnosed. A general history and examination, especially of the other cranial nerves, should not, of course, be omitted. Isolated palsies of extra-ocular muscles are, however, common—for example in dissemated sclerosis, myasthenia gravis (unilateral ptosis is also common and may be quite sudden in onset: variability is important) and diabetus mellitus (peripheral neuropathy which often involves the trigeminal nerve also). In the attempt to locate and diagnose the lesion, it is most useful to trace the nerve, in imagination, from the muscle supplied (hereditary ocular myopathy may, rarely, occur; "thyrotropic" infiltration with lymphocytes and fibrous tissue is usually accompanied by proptosis) to its nucleus and consider what neoplastic, inflammatory, vascular etc. causes may affect it en route.

### *Ocular Torticollis*

A child or an adult may have an abnormal posture of the head for a variety [illegible] reasons, including "fibrosis" of a sternomastoid, abnormalities of the vertebrae e[illegible] One rather uncommon cause is a congenital weakness of an extra-ocular musc[illegible] usually a superior oblique or a superior rectus. The cause is unknown, but bir[illegible] injury is often blamed; some cases have been shown to be due to poor developmen[illegible] differentiation of the muscle or to abnormal fibrous bands which limit movement [illegible] an eye. A child with such a disability, if it affects a muscle which moves the eye up [illegible] down, may be able to overcome his vertical diplopia and build up good stereoscop[illegible] reflexes by (a) holding his head backwards or forwards to relieve a paretic elevator [illegible] depressor respectively, (b) tilting his head over towards the shoulder on the same si[illegible] as the eye with the higher *image* and (c) turning his head so that the responsibil[illegible] for elevating and depressing the affected eye is transferred to another set of muscl[illegible] Which of these tricks is employed by the patient will depend on the affected musc[illegible] A right lateral rectus palsy will result merely in a turn of the head to the right. The co[illegible] mon congenital paresis of a right superior rectus muscle will be compensated by [illegible] backward tilt of the head, a turn to the right to transfer the onus for elevating a[illegible] depressing the eye to the oblique muscles (which act mainly on the adducted e[illegible] and finally, a tilt towards the right shoulder. This last element in the posture has t[illegible] advantages: (a) by postural and vestibular reflexes it compensates for the abnorm[illegible] height of the right eye's image and (b) it compensates for the outward wheel-rotati[illegible] of the *other* eye produced by the synergist of the *right* superior rectus, which is t[illegible] *left* inferior oblique. In adult life, compensation may sometimes begin to break do[illegible] and the patient complain of intermittent vertical diplopia. Photographs taken in chil[illegible] hood may show that he has had a head tilt all his life—and may be the only foundati[illegible] for an accusation of superciliousness (elevator palsy with nose in the air) or suspicio[illegible] ness (depressor palsy with head bent forwards, eyes looking "under his brows[illegible]

### *Treatment*

If the symptoms are severe enough and there is no chance of further spontane[illegible] improvement (e.g. 6–9 months after a head injury) then operation may be consider[illegible] Ophthalmologists are not usually enthusiastic in transplanting the outer (or inn[illegible] halves of the superior and inferior recti to the insertion of a paralysed lateral rec[illegible] muscle though some good results have been reported; a weakening, by recession [illegible] its insertion, of the medial rectus on the same side is usually more certain to produ[illegible] improvement. In such a case, recession of the *other* eye's medial rectus (a synerg[illegible] of the opposite lateral rectus) will reduce the area of gaze in which diplopia is suffer[illegible] for it is over-acting in response to the redoubled efforts of the brain to force the e[illegible] to look to the side of the paralysed muscle. Even in a complicated case of unilate[illegible] third cranial nerve palsy improvement can be achieved; if ptosis is effectively eli[illegible] nating the diplopia, however, operation may be best avoided. Considerable impro[illegible] ment in symptoms due to a paresis of a superior oblique muscle can be obtained [illegible] myectomy of the inferior oblique on the same side, along with recession of the ov[illegible] acting contralateral synergist, the inferior rectus.

### II CONCOMITANT SQUINT

This occurs especially in children and the main factor in the very common "acco[illegible] modative concomitant convergent squint" is hypermetropia. Another common t[illegible] is the weakness of convergence (i.e. latent, or intermittently manifest, divergent squ[illegible] for near objects) which often affects the elderly presbyope or the student hard at [illegible] last-minute reading for an examination. In either case, after the history of age [illegible] onset, intermittency etc. has been obtained, it is vital to check that there is a full ra[illegible] of movement in each eye separately before concomitance is accepted.

FIG. 1. *Upper.* On a small scale, emmetropic eyes look at an infinitely distant object. Visual axes almost parallel.

*Lower.* On a larger scale, emmetropic eyes look at a near object by (*a*) accommodating to bring divergent rays to a focus on the maculae and (*b*) converging by a proportionate amount to avoid diplopia.

A typical symptom in accommodative squint in children is that the squint is worse or even only present, when the child looks at a near object. The following is a brief account of the mechanism:

Normal eyes are emmetropic, i.e. no accommodation is required for distance. In order that an eye can produce a clear image of a near object, the ciliary muscle must contract (accommodation) which makes the curvature of the surface of the crystalline lens greater. In addition, if diplopia for near objects is to be avoided, each eye must be turned nasal-wards ("converged") enough to make the two visual axes intersect [illegible] the object. See Fig. 1. Now, the brain has a mechanism for "gearing" accommodation to convergence in *normal* eyes. But if the patient is born with hypermetropic eyes (i.e. his lens system is too "weak" for the length of his eyeball) he must accommodate *even for distant* objects and, therefore, to a greater degree than normal for near. Unfortunately, the gearing mechanism will therefore tend to produce over-convergence, i.e. a convergent squint, unless the harmonic reflex can alter the ratio of the gears. Fortunately it often succeeds, because all hypermetropes do not squint.

Many cases quickly develop a constant squint, however. If the two eyes are equally hypermetropic and see equally well, the child may choose to "alternate" i.e. use either eye indiscriminately, allowing the other to converge. But if one is slightly more hypermetropic than the other or has astigmatism or some other abnormality causing blurred vision (e.g. chorioditis due to toxoplasmosis in the macular region: note the implication here that a convergent squint may be a presenting symptom of unilateral disease of the eye) then the child will use the better eye consistently and neglect the other, which becomes permanently convergent. The danger now is that the child, suffering diplopia, suppresses the image from the squinting eye (cf. the practised microscopist who can keep both eyes open, suppressing the eye not in use), *a condition which in children quickly becomes permanent*, hence amblyopia ex anopsia. There follow then two reasons for *early* investigation of strabismus i.e. as soon as it has appeared. Early treatment of hypermetropia with spectacles (even a child of 18 months may be induced to wear spectacles) may "cure" the squint and prevent the need for operation. The sooner an amblyopic eye is treated (a) with spectacles if necessary, and (b) by occlusion of the other eye, the less chance it has of suffering permanent impairment of vision. If a convergent squint still remains after that treatment then recession of the medical rectus/recti is usually done, sometimes with resection of the lateral recti. Thus, two or more operations at different times may well be required.

The presbyope must almost invariably wear glasses for reading in order to replace the deficiency in accommodation. In addition, many develop weakness of convergence, with a complaint not only of blurring of individual letters on a page but that intermittently, "the letters run into each other" i.e. horizontal diplopia. Mild degrees respond to convergence exercises which consist in their simplest form, of looking [illegible] say, some small print (wearing the reading correction) and repeatedly approximating it to the eyes. Such an exercise should be done for 5 minutes three times daily: a week usually is sufficient. More severe degrees may require the incorporation of base-[illegible] prisms in the reading glasses.

Strabismus, in the widest sense of the term, is common and affects patients of [illegible] ages. Treatment is a matter of constant careful supervision, in which operations, [illegible] required, are merely incidents.

## Figures and Tables

**Fig. 1. f1:**
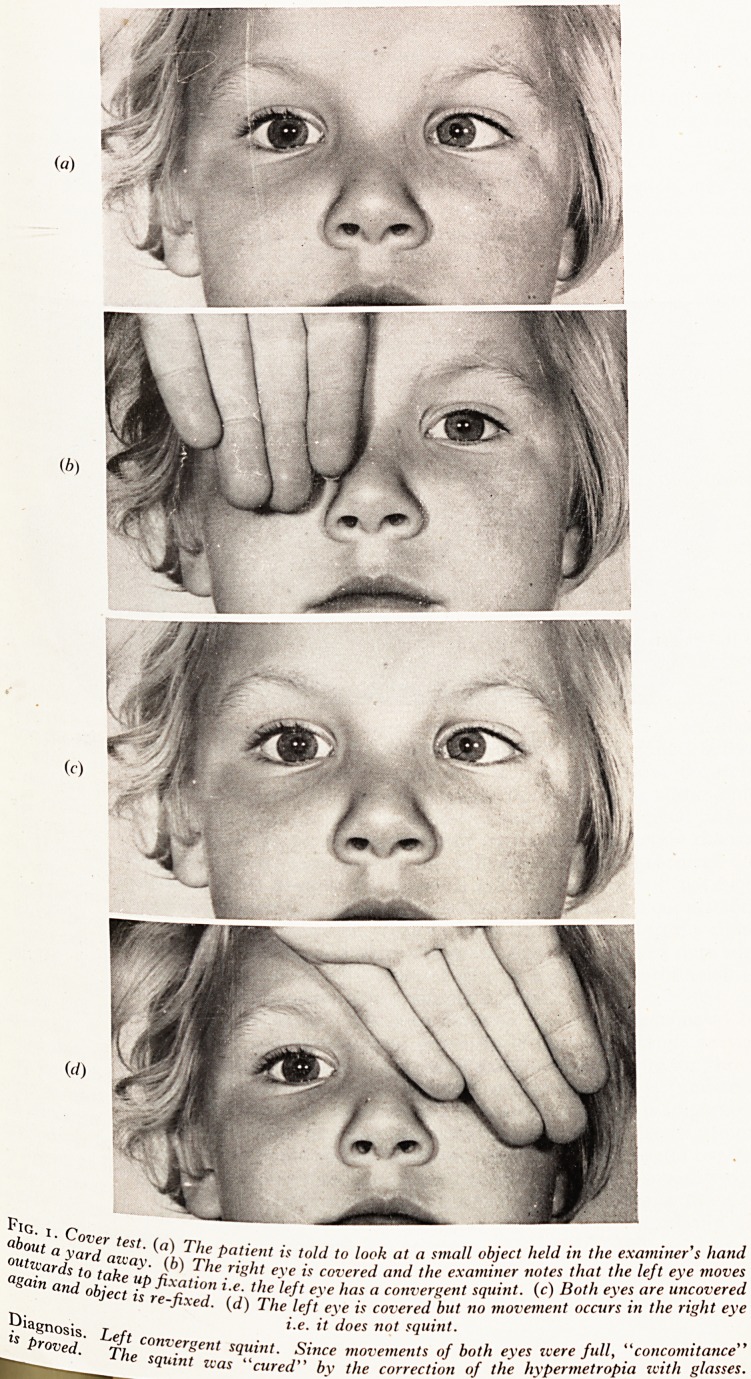


**Fig. 2. f2:**
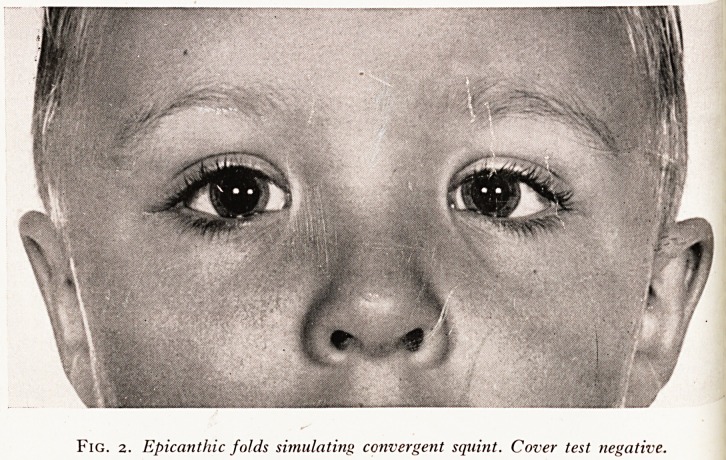


**Fig. 1. f3:**